# Comparison of different neuromuscular facilitation techniques and conventional physiotherapy in knee osteoarthritis

**DOI:** 10.3906/sag-2101-298

**Published:** 2021-09-24

**Authors:** Ayşenur GÖKŞEN, Filiz CAN, Seval YILMAZ, Feza KORKUSUZ

**Affiliations:** 1 Department of Physiotherapy and Rehabilitation, Faculty of Health Sciences, Karamanoğlu Mehmetbey University, Karaman, Turkey; 2 Department of Orthopedic Rehabilitation, Faculty of Physical Therapy and Rehabilitation, Hacettepe University, Ankara, Turkey; 3 Department of Physiotherapy and Rehabilitation, Faculty of Health Sciences, Yalova University, Yalova, Turkey; 4 Department of Sports Medicine, Faculty of Medicine, Hacettepe University, Ankara, Turkey

**Keywords:** Osteoarthritis, knee, exercise, proprioception

## Abstract

**Background/aim:**

This study was conducted to compare the effects of conventional physiotherapy and two different ‘proprioceptive neuromuscular facilitation’ (PNF) techniques on knee muscle strength, knee muscular endurance, and proprioception in knee osteoarthritis (KOA).

**Materials and methods:**

The study included 35 patients between the ages of 47 and 62 who were diagnosed with stage 1–2 KOA. The patients were divided into three groups with block randomization method as Repeated Stretching Group (N = 12) With Repeated Stretching Technique, Combined Isotonic Contraction Group (N = 11) With Combined Isotonic Contractions (CIC) Technique, And Conventional Physiotherapy Group (n = 12). PNF was applied to all patterns of the lower extremity in full pattern and patients in all groups were treated for 6 weeks, 3 days a week. Muscle strength, muscle endurance, and proprioception were evaluated with Biodex System Pro3 (Biodex Corp. Shirley NY, USA).

**Results:**

Knee extensor muscle strength showed more improvement at CIC group than the other groups, and CIC group showed more improvement compared to the conventional physiotherapy in terms of knee joint position sensation evaluated at 60° (p < 0.05).

**Conclusion:**

All methods were effective in patients with early-stage knee osteoarthritis; however, the most effective results were obtained by PNF using CIC technique.

## 1. Introduction

Proprioceptive neuromuscular facilitation (PNF) techniques are one of the exercise methods that strengthen muscles using neurophysiological mechanisms. PNF techniques could provide solutions for many problems such as muscle weakness, loss of proprioception, functional performance decrease that are seen in knee OA (KOA). PNF exercises include lots of strengthening exercise technique, which have different neurophysiological effects for lower extremity muscle. It is very important to choose the most appropriate PNF technique for the solution of problems that could be seen in OA. Technical choice is important in PNF method to obtain maximum benefit from the neurophysiological effects [1, 2] but the most suitable and effective PNF technique for knee OA is not known. In a systematic review that studies PNF techniques, it is reported that there is no study comparing the effectiveness of Repeated Stretching Technique and Combined Isotonic Contractions Technique on patients with KOA [3]. Also, there is not much study about PNF for musculoskeletal disease, including lower extremity, especially knee OA in the literature [3, 4]. This study contributes to literature in terms of using different PNF techniques that strengthens lower extremity muscles and effect of PNF on muscle endurance and proprioception, which is less studied in literature [3, 5–7]. Although PNF techniques use a high rate of receptors and proprioceptive pathways in muscles, tendons and joints, there are few studies in the literature that search the effect of PNF on proprioception [7]. The aim of this study is to investigate the effects of 6-week physiotherapy and rehabilitation programs that include two different ‘Proprioceptive Neuromuscular Facilitation’ (PNF) techniques which include repeated stretching technique and combined isotonic contractions technique and conventional physiotherapy program in patients with knee osteoarthritis on muscle strength, muscle endurance, and knee proprioception.

## 2. Material and methods

This study was planned as a prospective randomized controlled study, and the block randomization method was chosen as the randomization method [8]. Ethics committee approval with 2018/04-39 (KA-17112) decision number was received on 15.03.2018. Patients aged 45–65 years with a diagnosis of stage 1–2 knee OA were included in the study. Volunteers between 45–65 ages who have no hearing, vision and speech impediment, no meniscus and no ligament problems included the study. Patients with intra-articular contracture were excluded from the study. Patients who have any neurological or cardiopulmonary disease were excluded. All patients were divided into three treatment groups as Repeated Stretching Group, Combined Isotonic Contraction Group and Conventional Physiotherapy Group. Hot pack, ultrasound, isometric, and isotonic strengthening exercises for the hip and knee muscles, mini squatting and lunge exercises were applied in the conventional physiotherapy group. Hot pack, ultrasound, and exercises that include PNF techniques were applied to Repeated Stretching Group and Combined Isotonic Contraction Group. Repeated stretching technique and combined isotonic contraction technique were used in Repeated Stretching Group and Combined Isotonic Contraction Group, respectively. Additionally, “hold relax” technique was applied in both PNF groups. Full lower extremity patterns were applied to the all PNF groups. Patients in all three groups were treated 3 days a week for 6 weeks. Muscle strength, muscle endurance, and knee proprioception of whole patients were evaluated in pretreatment and posttreatment.

### 2.1. Muscle strength and muscular endurance

Isokinetic muscle strength and muscular endurance of M. Quadriceps Femoris and Hamstring muscle groups were evaluated concentrically by Biodex System Pro3 (Biodex Corp. Shirley NY, USA) isokinetic test dynamometer. The dynamometric test was performed in 5 repetitions at 60°/s angular velocity to evaluate muscle strength, and 20 repetitions at 180°/s angular velocity to evaluate muscular endurance. Thirty seconds of rest periods were given between the tests. Peak torque measurement was recorded as Newton meter (Nm). The total work during all repetitions was recorded as Joule (J) [9].

### 2.2. Proprioception

“Active position sense” was measured to evaluate knee proprioception by “Biodex System Pro3 Isokinetic System (Biodex Corp. Shirley NY, USA) at 30°, 45° and 60° as suggested in the literature [10–12]. Deviation from the target angle was recorded as the absolute angular error, regardless of whether the error was negative or positive. The test was repeated three times. Average of absolute angular errors generated by the patients in 3 repetitions at each test angle was processed as data.

### 2.3. Statistical analysis

Quadriceps Femoris muscle strength was chosen as the main outcome scale, since the application methods used in our study primarily focused on increasing muscle strength, and other parameters were expected to change depending on the increase in muscle strength [13]. Accordingly, with power analysis, when the significant difference between the 3 treatment groups is predicted to be 50% with 5% type 1 error, bidirectional hypothesis design is used to achieve 80% working power, ideally 10 for each group in this study. It was concluded that a total of 30 patients should be taken out of each patient [14, 15]. In order to increase the sensitivity in line with these power analysis results, the study was started with a total of 50 patients; however, the study was completed with 35 patients. The data obtained during the research process were analyzed with Jamovi 0.9.4.0 and SPSS 25 software. The conformity of the variables to the normal distribution was evaluated with Shapiro–Wilk; Nonparametric tests were used in statistical analyzes because the number of participants in the groups was low and the data did not conform to normal distribution. “Quantitative variables” obtained from the data, as median, 1st quartile, and 3rd quartile; “Qualitative variables”, on the other hand, were defined with numbers and percentages. Distributions of qualitative variables in independent groups were analyzed with the Chi-square test. The reason for using this test is to check whether the distribution of qualitative variables (e.g., sex) in the 3 groups is homogeneous. The aim here is to avoid the interference of a factor other than the intervention effect, which is the source of the difference in the groups in the response variable. Quantitative variables were tested with the Kruskal–Wallis test. Since the test statistic produced as a result of the Kruskal–Wallis test conforms to the Chi-square test distribution, Chi-square test results are also given in the findings to increase reliability. In cases where the Kruskal–Wallis test result was found to be significant, pairwise comparisons were made with the Dwass-Steel-Critchlow-Flinger test. Pre and posttreatment comparisons for quantitative variables were tested with the Wilcoxon signed-rank test. In cases where the number of dependent groups was greater than two, the Friedman test was used, and when it was significant, pairwise comparisons were made with the Durbin–Conover test. The significance level for all analyzes was determined as 5% (p < 0.05).

## 3. Results

A total of 35 patients (22 females, 13 males) between the ages of 47 and 62 with a diagnosis of stage 1–2 knee osteoarthritis were included to the study. The flow diagram of the study is shown in Figure. Baseline characteristics are shown in Table 1. As shown in Table 2 and Table 3, knee extensor muscle strength, knee flexor and extensor muscular endurance, knee joint position sensation that were evaluated at 45° improved in all treatment groups compared to pretreatment. Table 3 additionally shows that knee joint position sense improved in all angles in Repeated Stretching and Combined Isotonic Contraction groups. According to the isokinetic muscle strength evaluation after the treatment, there were statistically significant differences between the groups in terms of changes in the ratio of peak torque/body weight of knee extensors at 60°/s as shown in Table 2. According to pairwise comparisons, it was concluded that the improvement in knee extensor muscle strength in the Combined Isotonic Contraction Group was significantly higher than the change in the Conventional Physiotherapy Group as shown in Table 4. Table 2 shows that muscular endurances of knee extensor and knee flexor improved significantly in all groups. Changes in total work during knee flexion and knee extension in both PNF groups were found significantly greater than the change in the Conventional Treatment Group as a result of pairwise comparisons. Knee joint position sense was improved in both PNF groups at all angular velocities. In the Conventional Physiotherapy Group, a statistically significant improvement was observed only at 45°. When the groups were compared with each other with regard to knee joint position sensation, the change in the Combined Isotonic Contraction Group was found significantly higher (absolute degree) than the change in the Conventional Physiotherapy Group as shown in Table 5.

## 4. Discussion

According to the results we obtained from our study, the use of PNF method, which includes combined isotonic contractions technique, is more advantageous than other methods in terms of improving knee extensor muscle strength (p = 0.015**)**. In terms of improving muscular endurance, both PNF methods can be preferred to conventional physiotherapy method (p = 0.001). Lots of study in the literature focus on increasing flexibility and range of motion with the use of PNF techniques. In the literature, the use of PNF exercises to improve muscle strength and endurance is less [3, 5, 16]. In a systematic review study examining PNF techniques, it was reported that there was no study comparing the efficacy of the repeated stretching technique and the combined isotonic contractions technique on patients with OA [3]. Repeated stretching technique increases the number of motor units fired in the muscle by repeatedly stimulating the receptors of the relevant muscle. Combined isotonic contractions technique, which is another technique used for strengthening in our study, enables the improvement of muscle strength both concentrically, eccentrically and isometrically, by using the physiological mechanisms of the repeated stretching technique [2]. In the combined isotonic contractions technique, synchronous concentric, eccentric and isometric contraction of the muscles may lead to an increase in cortical responses, resulting in a greater increase in concentric muscle response and strength compared to other methods [17]. Therefore, combined isotonic contraction technique may have been more successful than repeated stretching technique in terms of increasing knee extensor muscle strength. Previous studies reported that when eccentric exercises are added to the concentric exercise program, a greater increase in muscle strength is obtained [18]. At the same time, the current literature suggests the combined application of concentric, eccentric and isometric exercises to maximize muscle strength [17–19]. In some studies, in the literature, improvement in muscle strength was achieved with the application of the PNF method, as in our study [20–22]. In some studies, unlike our study, improvement in muscle strength was not achieved with PNF training [23, 24]. The reason for this difference in the literature may be the technique used during PNF applications, application pattern, number of repetitions, and duration of application. Particularly, in the studies examining the effect of contract-relax and hold-relax technique on strengthening in the literature, the PNF method was not found to be effective on muscle strength [23, 25], because the main purpose of these techniques is to increase the range of motion and flexibility of a joint. In many studies using the technique of rhythmic stabilization, repeated stretching, and combined isotonic contractions, an improvement was observed in muscle strength [5, 21]. Based on the results of muscle strength evaluated isometrically in the study conducted by Pereira et al. [26], there was no difference in knee flexor muscle strength while a significant improvement was observed in knee extensor muscle strength in the group with PNF training compared to the conventional program. The results of their study support the results of our study. Independent of neuromuscular gains, it was predicted that such a result could occur due to the inconsistency of measurements with PNF exercise patterns. The improvement of knee extensor muscle strength may have resulted from the decrease in muscle coactivation due to reciprocal inhibition, one of the neurophysiological effects of training [2]. Strength increase can also improve due to supra-spinal neural adaptation outside the peripheral pathways. In fact, it may have improved muscle activation by giving the correct motor input rather than increasing strength [22]. Another theory is the improvement of knee extensor muscle strength due to the increase in proprioception [27]. Due to the proprioceptive response improved by PNF exercises, the neurostimulation threshold at the cortex level may be increased [22]. In the literature, there are a limited number of studies examining the effects of PNF on muscular endurance [5, 28]. It was argued that PNF training improved muscle strength rather than muscular endurance in the study conducted by Kofotolis et al. in 2005 [28], while it was concluded that PNF training improved muscular endurance in the study conducted by Kofotolis et al. in 2006 [5]. The results may have changed due to the difference in the patterns chosen in the studies, the variability of the patient group treated, the duration of the application, and the number of repetitions. It is known that low-intensity exercises performed up to the fatigue limit improve muscular endurance [26, 29]. PNF includes high-intensity exercise training with maximal resistance. For this reason, more studies are needed to examine the effects of PNF applications on muscular endurance. Proprioceptive neuromuscular facilitation (PNF) training mainly includes stimulation of joint receptors and improvement of proprioception. By facilitating or inhibiting neurological mechanisms, there is the regulation of movements, activation, or inhibition of muscles [2]. Despite this, studies examining the effect of PNF on proprioception are very few in the literature [7, 22, 26]. In a systematic review study conducted in 2019, it was reported that there is insufficient information about the effect of PNF on joint position [7]. Based on the results of our study, when PNF method is compared to the conventional method in terms of improving proprioception in patients with early-stage knee OA, the therapy method using the combined isotonic contractions technique was found to be more successful among the examined PNF methods than other methods. PNF method stimulates proprioceptive pathways more than conventional physiotherapy and makes progress even in knee joint angles where contact is much less. Joint position sensation, assessed at 60°, improved only in the group combined isotonic contraction group among the three groups after therapy, compared to pretherapy (p = 0.005). Joint position sensation, assessed at 60°, showed more improvement in the paired comparisons in the group combined isotonic contraction group compared to the group repeated stretching (p = 0.018). This indicates that PNF is superior to conventional physiotherapy at low angles where proprioceptive input is less and that the combined isotonic contractions technique is specifically more effective than PNF, even at angles where the proprioceptive input is slightly better. In other studies, in the literature, as in our study, the PNF method contributed to the improvement of knee joint position sensation [7, 20]. Although there are many factors affecting proprioception, the improvement in knee joint position with PNF training may have occurred due to the reduction of knee-related pain and increased muscle strength around the knee [30]. In the study of Hortobagyi et al., they concluded that knee extension muscle weakness negatively affected the proprioceptive sensation [31]. Our study concluded that the patients in the combined isotonic contraction group, which showed the most improvement in proprioception, also showed the most improvement in knee extensor muscle strength compared to the other groups. Similar to the studies in the literature, the improvement in proprioception may be affected by the improvement in muscle strength [20, 31]. The fact that the patellar tilt is high at 60° knee flexion angle in patients with knee osteoarthritis causes the patella not to fit well into the trochlear groove, thus reducing the contact that should be high at 60°, and consequently, a decrease in proprioceptive input [32]. For this reason, reducing patellar tilt and removing soft tissue tensions that cause patellar tilt can allow to reduce the error in joint position sensation that occurs in this angle. In our study, the joint position sensation evaluated at 60° was the angle that made the difference between the groups so as to support this information. As reported by Erduran et al. a significant difference in the knee joint position sensation at 60° knee flexion may have been due to the tension in the tissues around the knee [32]. Based on this view, if the problem stemming from soft tissues is solved, the knee joint position sensation evaluated at 60° may improve. When the groups in our study were compared with each other, knee joint position sensation evaluated at 60° showed the highest level of improvement in combined isotonic contraction group. According to this result, the best solution to soft tissue problems found in knee OA can be provided by the combined isotonic contractions technique, which includes both hold-relax, repeated stretching and concentric, eccentric and isometric contractions together. Thus, the joint position sensation can be increased by reducing soft tissue tensions, decreasing patellar tilt, and increasing contact. In conclusion, in our study in which two different PNF methods were compared to each other and to the conventional physiotherapy method in patients with early-stage knee OA, it was observed that all three methods had positive effects in terms of muscle strength, muscular endurance, and proprioception (joint position sensation). PNF was found to be more effective in terms of muscle strength, muscular endurance, and proprioception (joint position sensation). It has been concluded that the combined isotonic contractions technique, which also includes the repeated stretching technique in PNF, is more effective in improving proprioception (joint position sensation) and muscle strength in patients with early-stage knee OA. In conclusion, determining the deficiencies after a very detailed evaluation of patients with early-stage knee OA, and choosing the appropriate PNF techniques and patterns will increase the efficacy of physiotherapy and rehabilitation programs. The results of this study are very important in terms of revealing the clinical importance of the PNF technique, which is one of the most important techniques in physiotherapy and rehabilitation, but, recently, its clinical use is gradually decreasing with the introduction of new exercise types or equipment, causing researchers to question its clinical efficacy. Also, this study is very important in terms of showing the evidence-based results of a technique on which few scientific studies have been conducted, although it has been a frequently used method in clinical practice for years.

## Figures and Tables

**Figure:**
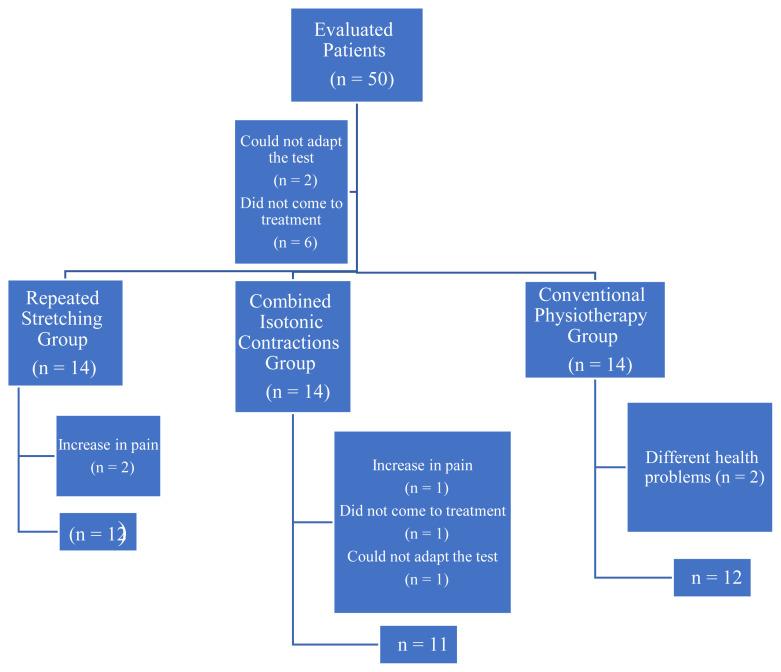
Flow chart of.

**Table 1:** Baseline characteristics of.

Characteristics		Repeated Stretching Group n = 12	Combined Isotonic Contraction Group n = 11	Conventional Physiotherapy Group n = 12	p value
		Mean±SD			
**Age**		53.5 ± 6.58	55.18 ± 7.29 (47–62) ± 7.29	54.33 ± 6.99 (48.5–61) ± 6.99	0.937
**Body mass index (kg/m** **^2^** **)**		30.41 ± 3.63	31.22 ± 3.69	30.61 ± 5.33	0.781
		**n (%)**			
**Sex**	**Female**	8 (66.7)	6 (54.5)	8 (66.7)	0.789
	**Male**	4 (33.3)	5 (45.5)	4 (33.3)	

n: number of patients; SD: Standard deviation; Age and Body mass index p values obtained using the Kruskal–Wallis test; Sex p values obtained using Chi-square test.

**Table 2:** Pretreatment and posttreatment muscle strength and muscular endurance results.

Isokinetic muscle strength and muscular endurance		Pretreatment	Posttreatment	Wilcoxon test	
		Median (1.–3. Quarter)	Median (1.–3. Quarter)	z	p value
**Repeated Stretching Group**	**Knee extensors peak torque/body weight ratio PT/BW (%) 60 °/sec**	93.4 (85.5–98.9)	114.15 (97–126.43)	−3.062	**0.002** *
	**Knee flexors peak torque/body weight ratio (PT/BW) (%) 60 °/sec**	45.4 (23.53–46)	48.75 (36.6–68.5)	−3.062	**0.002** *
	**Total work during knee flexion (J) 180°**	97 (38.05–153.5)	162 (95–279)	−3.061	**0.002** *
	**Total work during knee extension (J) 180°**	212.95 (162.5–615.85)	580 (425–1019.95)	−3.059	**0.002** *
**Combined Isotonic Contractions Group**	**Knee extensors peak torque/body weight ratio PT/BW (%) 60 °/sec**	84.6 (66.8–130.4)	110.5 (100–161.3)	−2.934	**0.003** *
	**Knee flexors peak torque/body weight ratio (PT/BW) (%) 60 °/sec**	48.4 (44.7–67.3)	55.3 (47.4–64)	−1.067	0.286
	**Total work during knee flexion (J) 180°**	257 (140.2–458.7)	400.3 (148.4–555.3)	−2.490	**0.013** *
	**Total work during knee extension (J) 180°**	887.4 (344.4–1081.7)	999.9 (798.2–1404.5)	−2.402	**0.016** *
**Conventional Physiotherapy Group**	**Knee extensors peak torque/body weight ratio PT/BW (%) 60°/s**	116.6 (51.25–141.85)	132.05 (71.95–149.5)	−2.512	**0.012** *
	**Knee flexors peak torque/body weight ratio (PT/BW) (%) 60°/s**	49 (25.78–57.6)	56.5 (46.6–63)	−2.788	**0.005** *
	**Total work during knee flexion (J) 180°**	177.2 (76.65–311.75)	181 (87.4–268.82)	−2.120	**0.034** *
	**Total work during knee extension (J) 180°**	752.3 (365.1–992.8)	755.55 (394.27–997.07)	−3.062	**0.002** *

* p: values obtained using the Wilcoxon test, p < 0.05; BW: Body weight; J: Joule; se: second.

**Table 3:** Knee joint position sense results between the groups pretreatment and posttreatment.

Knee joint position sense		Pretreatment	Posttreatment	Wilcoxon test	
Deviation Angles (°)		Median (1.–3. Quarter)	Median (1.–3. Quarter)	z	p value
**Repeated Stretching Group**	**Target angle 30°**	2.5 (1.78–5)	2 (1–2.75)	−2.117	**0.034** *
	**Target angle 45°**	4.5 (1–6.28)	2 (1–2)	−2.613	**0.009** *
	**Target angle 60°**	7 (3.75–13.25)	2 (2–4.5)	−1.962	**0.050** *
**Combined Isotonic Contractions Group**	**Target angle 30 °**	3 (1–9)	1 (0–4)	−2.716	**0.007** *
	**Target angle 45 °**	5 (2–7)	1 (0–1)	−2.677	**0.007** *
	**Target angle 60 °**	7 (3–12)	2 (0–5)	−2.812	**0.005** *
**Conventional Physiotherapy Group**	**Target angle 30 °**	3.5 (2–6)	3.5 (1–7)	−0.423	0.672
	**Target angle 45 °**	5 (2.5–11)	4 (0.25–5.75)	−2.025	**0.043** *
	**Target angle 60 °**	4 (3–7.75)	5 (1.5–11)	−0.179	0.858

* p: values obtained using the Wilcoxon test; p < 0.05.

**Table 4:** Comparison of improvement in isokinetic muscle strength and muscular endurance after treatment.

Groups		Repeated Stretching Group	Combined Isotonic Contractions Group	Conventional Physiotherapy Group	Kruskal–Wallis test	
		Median (1.–3. Quarter)	Median (1.–3. Quarter)	Median (1.–3. Quarter)	Chi-Square	p value
**Isokinetic Muscle Strength**	**Knee extensors peak torque/body weight ratio (PT/BW) (%) 60°/s**	22.95 (12.75–28.05)a,b	25 (15.4–33.2)^a^	8.1 (6.7–17.65)^b^	8.437	**0.015** *
	**Knee flexors peak torque/body weight ratio (PT/BW) (%) 60°/s**	11.25 (2–17.95)	6,9 (–5.6–10.3)	7.55 (4.9–12)	1.980	0.372
**Muscular Endurance**	**Total work during knee extension (J) 180°**	289.8 (183.55–435)^a^	134,4 (4–397,4) a	4.83 (3.15–6.6) b	15.57	**<0.001** ^**^
	**Total work during knee flexion (J) 180**°	65,75 (40–139.55)^a^	73.4 (4.1–238.5)^a^	4.99 (2.7–7.45)^b^	14.51	**<0.001** ^**^

PT: Peak torque;

* p: values obtained using the Kruskal–Wallis Test, p < 0.05;

**a-b-c:** the values shown with different letters in same line were statistically different (p < 0.05); BW: Body weight; J: Joule; s: second.

**Table 5:** Comparison of improvement of knee joint position sense between the groups.

Knee joint position sense	Repeated Stretching Group	Combined Isotonic Contractions Group	Conventional Physiotherapy Group	Kruskal–Wallis test	
Deviation Angles (°)	Median (1.–3. Quarter)	Median (1.–3. Quarter)	Median (1.–3. Quarter)	Chi-Square	p value
**Target Angle 30°**	−1 (−2 – 0.35)	−1 (−5 – 1)	−0.5 (−1.5 – 0)	2.644	0.267
**Target Angle 45°**	−3 (−4.35 – 1)	−2 (−6 – 1)	−1 (−4.5 – 0)	1.436	0.488
**Target Angle 60°**	−3 (−9.5 – 1)a,b	−5 (−7 – 1)^a^	0 (−2 – 1)^b^	8.054	**0.018** *

* p: values obtained using the Kruskal–Wallis Test, p <0.05;

**a-b-c:** the values shown with different letters in same line were statistically different (p<0.05).
